# Severe Babesiosis in Immunocompetent Man, Spain, 2011

**DOI:** 10.3201/eid2004.131409

**Published:** 2014-04

**Authors:** Luis M. Gonzalez, Susana Rojo, Fernando Gonzalez-Camacho, Daniel Luque, Cheryl A. Lobo, Estrella Montero

**Affiliations:** Centro Nacional de Microbiologia, Majadahonda, Spain (L.-M. Gonzalez, F. Gonzalez-Camacho, D. Luque, E. Montero);; Hospital Universitario Central de Asturias, Asturias, Spain (S. Rojo);; New York Blood Center, New York, New York, USA (C.A. Lobo)

**Keywords:** *Babesia divergens*, human babesiosis, immunocompetent, treatment failure, tick-borne, vector-borne infections, vector, protozoan, Ixodes, parasite, zoonoses

**To the Editor:** Babesiosis, a malaria-like illness, is transmitted through *Ixodes* ticks by the zoonotic parasites, *Babesia* spp. In humans, these parasites are transferred from mammalian animal reservoirs, and the rate of infection in humans is increasing. Babesiosis also potentially threatens the blood supply because asymptomatic infections in humans are common; such infections can be life-threatening in some recipients (*1*). Most human infection is caused by *B. microti*, but babesiosis caused by *B. divergens*, *B. duncani*, and *B. venatorum* has been reported.

Human babesiosis can be clinically silent or progress to a fulminant malaria-like disease. The infection resolves spontaneously or after treatment with azithromycin/atovaquone or clindamycin/quinine. However, immunocompromised patients may respond suboptimally to these drug regimens (*2*). Given the death rate associated with babesiosis, no treatment is fully satisfactory (*3*). Infection with *B. divergens* is particularly problematic and is associated with a high death rate in splenectomized or immunocompromised patients (*3*). In Europe, sporadic cases of babesiosis have also been reported in immunocompetent persons (*4*).

In October 2011, a 46-year-old man whose spleen was intact was hospitalized after 3 days of fever, severe abdominal pain, jaundice, and black and red deposits in his urine. The man lived in a rural area in Asturias, Spain, where he was employed as a forest ranger. He reported that he removed ticks from his dogs.

Laboratory findings included hemoglobin 12.3 g/dL (reference range 13.8–17.2 g/dL); creatinine 1.52 mg/dL (reference range 0.7–1.3 mg/dL); total and direct/conjugated bilirubin 18.4 and 12.8 mg/dL (reference ranges total 0.3–1.9 mg/dL; direct/conjugated 0–0.3 mg/dL), lactate dehydrogenase 822 IU/L (reference range 105–333 IU/L; and showed thrombopenia, low haptoglobina, and hematuria. A value of 35% CD4+ T cells (reference range 30%–60%) indicated normal immune status. Results of serologic tests for hepatitis; HIV; and *Bartonella*, *Brucella*, *Leishmania*, *Leptospira*, and *Borrelia* spp. and of blood cultures were negative. Abdominal ultrasound scan revealed mild hepatomegaly and cortical echogenicity compatible with acute kidney failure. Howell-Jolly bodies were identified in blood, and functional splenic studies were conducted. Scintigraphic parameters showed a normal deposit of radioactive hepato-splenic material, compatible with a normal-sized spleen of 13.4 cm.

Giemsa-stained blood smears showed intra-erythrocytic parasites, mainly observed in the 2-celled dividing pyriform stage, leading to the diagnosis of babesiosis with a parasitemia level of 10% (Figure). The complete *B. divergens* 18S rRNA gene was amplified from the patient’s blood (*5*), and the nucleotide sequencing (GenBank accession no. KF533077) showed 100% homology with *B. divergens* human strains (GenBank accession nos. FJ944822 and FJ944823) (*5*) and with 2 babesiosis cases reported previously (*4*). Indirect immunofluorescent assays of *B. divergens* cultures showed specific antibodies against *B. divergens* in the patient’s serum (Technical Appendix Figure). The patient was treated with 650 mg oral quinine every 8 hours and 600 mg intravenous clindamycin every 6 hours. The parasitemia diminished gradually and resolved 10 days later, but the hemolytic anemia remained severe, as evidenced by hemoglobin of 7.2 g/dL.

**Figure:**
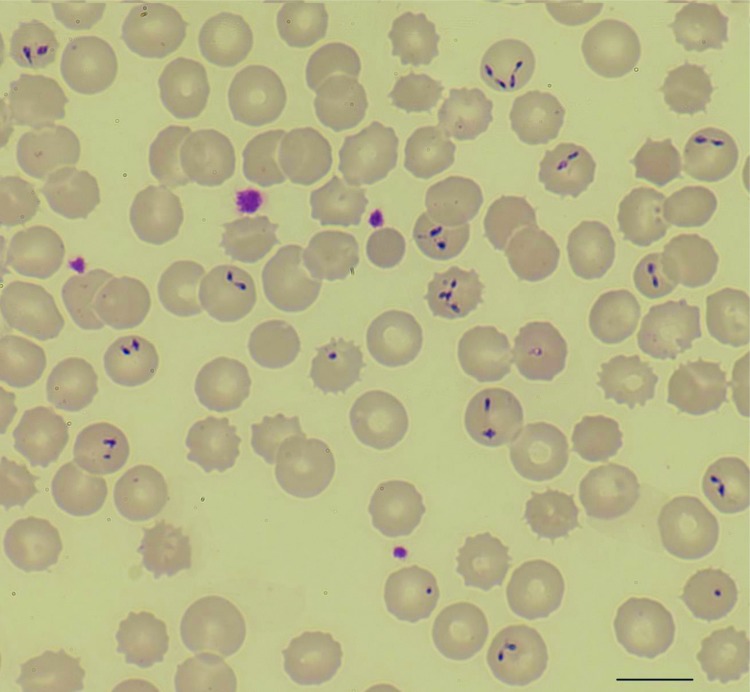
Photomicrography of a Giemsa-stained thin film of a 46-year-old man showing *Babesia divergens*. Double pear-shaped intraerythrocytic parasites are indicated by arrows. Slides were examined with a Nikon microscope at 60× magnification. Scale bar indicates 500 nm.

The man’s illness unexpectedly relapsed on day 18 after treatment. His reticulocyte count was elevated, and parasites were once again detected in blood samples. Thus, treatment was changed to a combination of atovaquone/proguanil 250/100 mg administered every 8 hours plus azithromycin 500 mg every 24 hours. Two weeks later, the patient’s hemoglobin was 8.7 g/dL, and no parasites were detectable by microscopy. The treatment was extended for an additional 5 weeks, and the patient was free of parasites on subsequent visits.

We have described what appears to be the third case of human babesiosis in nonsplenectomized patients in Europe. Human presence in tick, cattle, and domestic animal habitats could be responsible for this case. Martinot et al. (*4*) earlier pointed out that in Europe, babesiosis can also occur in persons with intact spleens. A combination of clindamycin and quinine is the recommended treatment of severe babesiosis (*2*,*3*). However, in this case, the recommended therapy failed, and therapy was switched to atovaquone/proguanil plus azithromycin. Other case reports have also related failure, ineffectiveness, adverse reaction, or persistent and relapsing babesiosis to clindamycin and quinine treatment in splenectomized patients infected by *B. divergens* or *B. microti* (*3**,**6**–**9*) or suspected *B. microti* drug resistance in immunocompromised patients (*2*). The recently sequenced *B. microti* genome reveals absence of proteases necessary to digest host hemoglobin and hemozoin formation by the parasite; this absence may explain the ineffectiveness of chloroquine, and perhaps other compounds of the aminoquinoline family used in babesiosis therapy (*10*).

This clinical case report, together with the failure of clindamycin and quinine to successfully eliminate the parasite *Babesia*, again opens the debate about the limitations of conventional treatment for severe human babesisosis in immunocompetent and immunocompromised patients. The capability of *Babesia* spp. to invade erythrocytes is the key step of the disease process. Focusing on *Babesia* spp. molecules involved in the invasion steps may offer new targets for the development of new prophylaxis and treatment for human babesiosis.

Technical Appendix Progress of immunofluorescent assays of human erythrocyte cultures in identifying *Babesia* spp.
