# Supplementary material for: Severe Babesiosis in Immunocompetent Man, Spain, 2011
Source: Emerg Infect Dis. 2014 Apr;20(4):724–6. doi: 10.3201/eid2004.131409 (PMC3966382; doi:10.3201/eid2004.131409)
Supplement: Technical Appendix — Progress of immunofluorescent assays of human erythrocyte cultures in identifying Babesia spp. [file 13-1409-Techapp-s1.pdf]

Article DOI: 10:3201/eid2004.131409

Severe Babesiosis in Immunocompetent Man, Spain, 2011

Technical Appendix

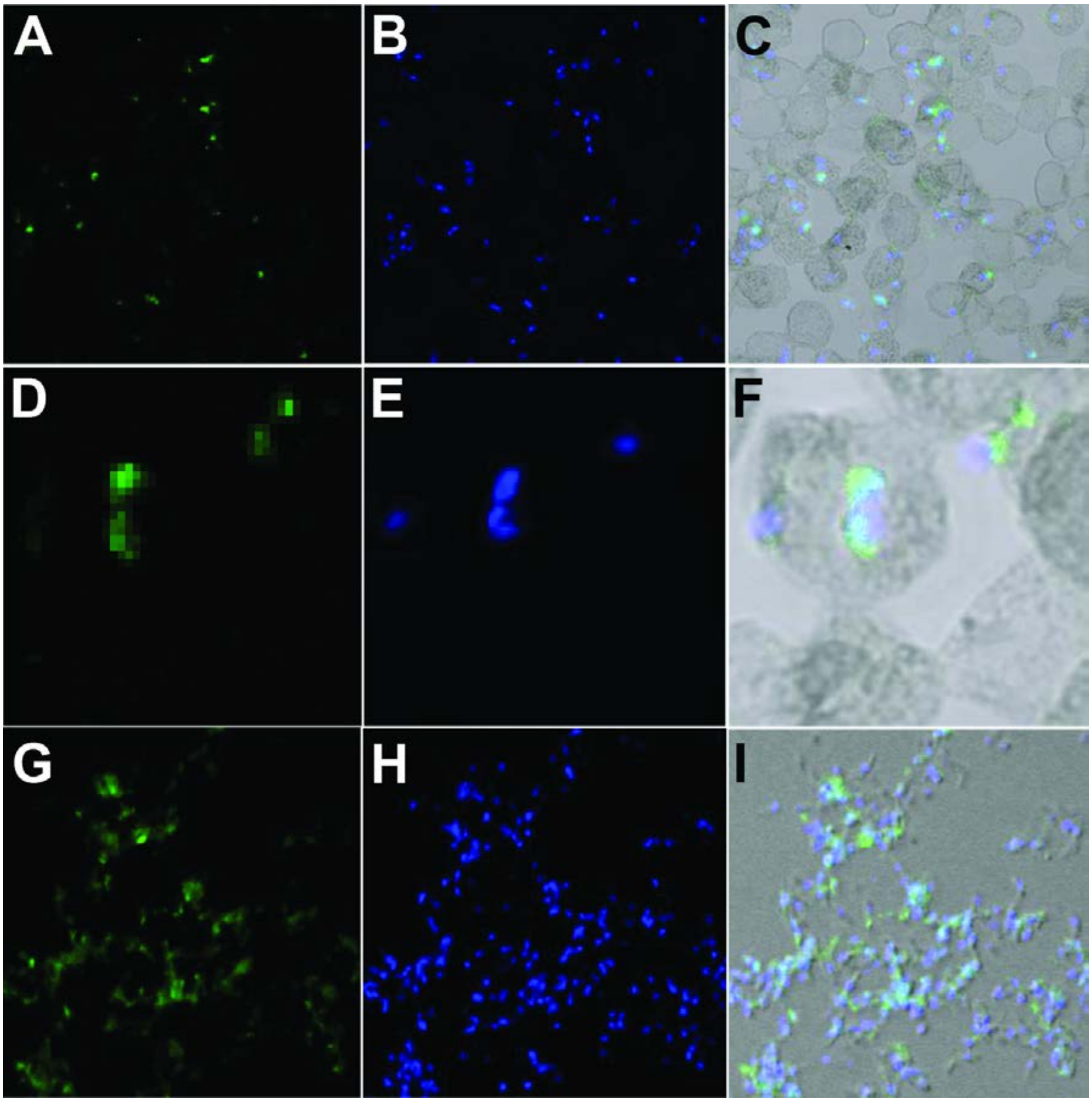

Technical Appendix Figure. Immunofluorescent assays of intra-erythrocytic and free parasites were performed by using antibodies and *Babesia divergens* smears of a 46-year-old man that were prepared from in vitro human erythrocyte cultures (Bd Rouen 1986 strain). Bound antibody was detected by using fluorescein isothiocyanate-conjugated anti–human IgG. The preparations were counterstained with DAPI and examined by confocal microscopy. Samples were laser-stimulated at 488 and 405 nm. Intraerythrocytic parasites probed with patient IgG are shown in panels A and B and isolated free extracellular merozoites in panel C. Panels D, E, and F show the DNA of these same parasites stained with DAPI. Panels G, H, and I show both images overlaid. The immunofluorescent assays, using serum from healthy volunteers, were negative (data not shown).
